# Optimization of biodegradable paper cup packaging coated with whey protein isolate and rice bran wax as potential popcorn package

**DOI:** 10.1002/fsn3.2628

**Published:** 2021-10-22

**Authors:** Sedigheh Ganjizadeh Zavareh, Majid Javanmard Dakheli, Behjat Tajeddin

**Affiliations:** ^1^ Medical Sciences Branch Department of Food Industry Islamic Azad University Tehran Iran; ^2^ Department of Chemical Engineering Iranian Research Organization for Science & Technology (IROST) Tehran Iran; ^3^ Packaging Engineering Agricultural Engineering Research Institute Agricultural Research, Education and Extension Organization (AREEO) Karaj Iran

**Keywords:** coating, paper cup, RSM, snacks, WVTR

## Abstract

Biodegradable paper cups coated with rice bran wax and whey protein isolate were designed to package popcorn. Coatings with different concentrations of whey protein isolate (5.5, 7.75, and 10% w/v) and rice bran wax (0.2, 0.4, and 0.6% w/v) were applied on the outer surface of the paper cups. Thickness, color changes, Young's modulus and tensile strength, water vapor transmission rate (WVTR) of the coated and uncoated cups, and also popcorns properties (pH, texture, and sensory properties) were evaluated. Water vapor transmission rate, Young's modulus, thickness, total color change index, and tensile strength of coated cups with the optimal coating formulation was 19.785 (g/m^2^ day), 11.810 (MPa), 276.583 (µm), 1.839, and 11.222 (MPa), respectively. The results showed that paper cup coating increased thickness and yellowness and reduced the brightness, Young's modulus, and WVTR. Coating had a positive effect on the pH and texture of popcorns packaged in coated cups than samples packed in uncoated cups (*p* < .05). With increasing storage time due to moisture absorption, popcorn changes from crisp to viscoelastic and increases tissue firmness (*p* < .05). Popcorns’ taste in uncoated cups had gained significantly lower scores by panelists compared with the samples packed in coated cups. There was a significant decrease in the general acceptance of popcorn during storage and also the type of coating used in the packaging cup (*p* < .05). Storage time and type of coating showed no significant effect on the moisture content, odor, and appearance of popcorn. In sensory evaluation, the coated packaging increased the taste, no difference in odor and appearance, and increased the overall acceptance of popcorn compared to the sample in uncoated cups. In general, the results showed that paper cup coating could be a new approach for barrier property improvement in paper‐based food packaging and extending the shelf life of the products.

## Graphical abstract

Biodegradable packaging is one of the best types of packaging that wishes not to harm the environment. Paper packaging coated with materials, such as rice bran wax and whey protein isolate, both of which are food waste, can improve paper color and resistance, and provide a good moisture barrier. This type of coating can be used in paper packaging of food such as snacks, noodle packaging, semi‐prepared foods, etc. This type of packaging can also maintain the physicochemical and sensory properties of food for a long time. Also, due to the edible nature of this type of coating, this coating can be used inside the package so that the printability of the packaging surface is not affected.

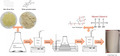

## 1 INTRODUCTION

One of the most important issues in food packaging is maintaining quality, increasing safety, and increasing the shelf life of food products (Jahdkaran et al., 2021). Plastic packaging is widely used in the food industry due to its low cost, easy processing, and availability (Agrillo et al., 2019; Mousavian et al., 2021). Petroleum‐based synthetic polymers are used in a variety of packaging. Increased use of petroleum polymers due to their nondegradability causes environmental problems as well as increasing dependence on petroleum products (Salwa et al., 2019). Today, paper and cardboard are the most popular types of naturally degradable material for food packaging. In terms of structural composition, paper is less complex than plastic, and therefore, migration (transfer of materials from packaging to food) is expected to be less than similar plastic samples. One of the most important problems in the use of paper and cardboard in food packaging is the early spoilage of food due to the permeability of this type of packaging (Hadaegh et al., 2014).

Edible coating is the simplest way to increase the functional properties of paper. Active compounds, such as polyethylene, wax, PVDC, etc., can be used as solvents, water‐based dispersions, or as solid melts. In general, the formation of the coating depends on several factors such as the type of solvent used, the temperature of the coating and drying, and the drying speed (Coles et al., 2003). Synthetic polymers as packaging material could be replaced by some agricultural wastes (Jawaid et al., 2017).

Rice bran wax is one of these agricultural wastes. It is one of the oldest coatings used on paper (Hadaegh et al., 2014). Real waxes belong to the group of simple lipids, and chemically, the esters of fatty acids and the monohydric alcohols that make them both have high molecular weights (Hamedi, 2008). Rice bran wax is a vegetable, hard and crystalline wax that is taken from rice bran and is a by‐product of rice mills. The main source of this wax is East Asia. Rice bran wax is hydrogenated from raw rice bran oil and is obtained in the waxing stage (Dassanayake et al., 2009). This wax is mainly composed of high molecular weight monoesters C64‐C46. Also, is used in nonfood applications including cosmetics, in the textile industry as a lubricant, machine‐building industry, etc. (Carlson, 2017).

In addition, whey is a natural waste of the dairy industry. In fact, it is a by‐product of cheese factories and with its protein and sugar compounds, it is a strong pollutant with a tendency to absorb high oxygen at the rate of 35–45 kg/L, which if not properly controlled can cause environmental problems (Vahedi et al., 2015). Whey is a liquid that is produced during the process of coagulation of milk in cheese production. Whey, or whey protein, is separated from the curd during the cheese‐making process and contains about half of the solids in whole milk. Whey protein isolates are the result of membrane separation processes such as ultrafiltration and diafiltration (Early, 1998). Whey protein isolate (WPI) is used in infant formulas, functional foods, and snacks due to its high nutritional value and good functional properties such as solubility, foaming, bonding, jelly, and emulsifying (Li et al., 2021). Numerous studies have been conducted on the types of coatings and their effects on paper permeability. However, coating with rice bran wax and whey protein isolate for popcorn paper packaging has been done for the first time in this research.

Popcorn, has been a delightful snack for centuries and has gained considerable commercial importance today. By heating the corn and reaching the appropriate temperature, it expands with an explosive sound and its volume reaches up to 30 times the volume of the main corn (Sweley et al., 2013). The primary packaging system for the retail sale of popcorn has been flexible bags made of polyethylene. Despite the superiority of metallized laminate which offers lower moisture vapor and oxygen transmission, clear polyethylene film is still used for popcorn packaging (Plimpton, 1984). Popcorn is low in moisture content and high in oils and other content susceptible to oxidizing. Ideal packaging for popcorn would exclude oxygen, moisture, and light.

Therefore, in this study, a blend of rice bran wax and whey protein isolate is used for coating the outer surface of paper cups as potential popcorn packaging. The effects of coating on the physical, mechanical, and surface properties of paper cups and then the effects of the packaging on the physicochemical properties of packed popcorn were evaluated.

## 2 MATERIALS AND METHODS

Unprinted paper cups were prepared from the Baba Salman region (Shahriar, Iran). Popcorn grain (*Zea mays* var. Everta), rice bran wax, whey protein isolate, Tween80, glycerin, and all laboratory materials were purchased from a local market (Karaj, Iran), Halal Everyday, Arla, DNAbiotech, Farabi, and Merck, respectively.

### 2.1 Coating solution preparing

Coating solution with different concentrations of whey protein isolate was prepared according to Table 1 in 100 ml of deionized water with a pH of 7.3. For better solubility of the protein in water, the mixture was stirred at 750 rpm for 15 min using a magnetic stirrer (Heidolph lnstruments Model D‐91126). The resulting solution was placed in a hot water bath (Rost Frei) at 90°C for 30 min to denature the protein. The resulting solution was then placed in a magnetic stirrer at 500 rpm at a set temperature of 90°C to maintain the temperature. Then, the weighed rice bran wax was added to the above solution according to Table 1 4 g of Tween 80 as an emulsifier was added dropwise, and after the wax was completely dissolved, finally 3 g of glycerin was added as a plasticizer (Hassani et al., 2010).

**TABLE 1:** Experimental design matrix with actual and predicted values of response variables

Test unit	Space Type	Actual values independent variables (coded values)		Observed values Response variables					Predicted values Response variables				
		Rice Bran Wax (g)	Whey Protein Isolate (g)	Thickness (µm)	∆E	Young's Modulus (MPa)	Tensile Strength (MPa(	WVTR (g/m^2^ day)	Thickness (µm)	∆E	Young's Modulus )MPa(	Tensile Strength )MPa(	WVTR (g/m^2^ day)
1	Factorial	(−1) 0.2	(−1) 5.5	269	1.37	13.68	12.53	30.13	270.083	1.94022	13.707	12.4871	30.58
2	Factorial	(+1) 0.6	(−1) 5.5	277	1.87	11.73	11.09	19.47	281.667	8.73456	11.2826	10.744	25.3518
3	Factorial	(−1) 0.2	(+1) 10	274	2.57	12.02	12.13	35.47	274	1.22123	13.066	12.3674	32.4384
4	Factorial	(+1) 0.6	(+1) 10	293	11.84	10.91	10.4	23.8	279	4.32474	12.0916	11.1884	22.8011
5	Axial	(−1) 0.2	(0) 7.75	275	2.66	13.12	12.19	32.66	286	5.50456	11.5893	10.9574	21.1384
6	Axial	(+1) 0.6	(0) 7.75	284	5.74	11.75	11.13	21.09	279	4.32474	12.0916	11.1884	22.8011
7	Axial	(0) 0.4	(−1) 5.5	273	3.39	12.63	11.47	21.96	291.417	12.1069	10.9904	10.4405	23.4366
8	Axial	(0) 0.4	(+1) 10	280	9.87	11.39	10.65	24.76	273.917	3.43855	12.047	11.9955	35.2416
9	Center	(0) 0.4	(0) 7.75	279	3.73	12.18	11.02	22.85	276.583	1.83855	11.8104	11.2221	19.785
10	Center	(0) 0.4	(0) 7.75	277	4.12	12.01	11.36	22.45	279	4.32474	12.0916	11.1884	22.8011
11	Center	(0) 0.4	(0) 7.75	282	3.45	11.87	11.19	22.93	272.333	2.85123	12.5226	11.3807	21.1951

### 2.2 Paper cups coating for popcorn packaging

The above obtained coating solutions were coated by immersion on the outer surface of paper cups after cooling at room temperature. The coated cups were kept at room temperature for 3 days to dry completely. After performing physical and mechanical tests on the cups and statistical analysis, the optimal concentration for coating the cups was determined. Then, to further investigate the optimal concentration of the coating and its effect on the storage rate of popcorn, 40 g of popcorn was poured into coated cups with rice bran wax and whey protein isolate, and cups without coating were selected as control. All cups were then capped and stored for three 14‐day periods in a laboratory environment with a temperature of 25°C and a relative humidity of 50%.

### 2.3 Test methods

#### 2.3.1 Thickness of the coated cup

To perform the thickness test, a digital micrometer (Mitutoyo) with an accuracy of 0.001 mm was used in six random positions on the coated cups’ wall. The maximum opening of the micrometer jaws in this device is 25 mm. The number was read on the device and the obtained numbers were converted to microns to facilitate the process of data analysis (Javanmard & Golestan, 2010).

#### 2.3.2 Color changes

The color of the coated cups was measured using a Hunter lab colorimeter (Model D25‐9000). Food color is typically measured in the L*, a*, and b* systems. L* a* b* or CIE Lab color space is an international standard for color measurement introduced by the International Lighting Commission in 1976. L* is an indicator of brightness and varies between 0 and 100, and the parameters a* (from green to red) and b* (from blue to yellow) are color components that vary between −120 and 120 (Yaqubi Surah et al., 2013).

To perform this test, the device was first calibrated using white and black tiles. The cups were placed in the machine and the factors L* (brightness or brightness of the sample), a* (red to green), and b* (yellow to blue) were measured. In addition to L*, a*, and b*, the amount of color change of the samples (∆E) was determined and reported using Equation 1.

(1) $$\Delta E=\sqrt{{\left(L^{*}‐L_{0}^{*}\right)}^{2}+{\left(a^{*}‐a_{0}^{*}\right)}^{2}+{\left(b^{*}‐b_{0}^{*}\right)}^{2}}$$

*L* ^*^_0_, *a* ^*^_0_, and *b* ^*^_0_ are the color parameters of the uncoated cup sample, and L*, a*, and b* are the color parameters of the coated cup samples.

#### 2.3.3 Mechanical properties

Mechanical test evaluation was performed using a Geotech testing machine (GT‐TCS‐2000 model). The coated paper cup was cut into strips 150 mm long and 15 mm wide. Then the strip cut by special jaws was fixed inside the machine. Jaws were spaced 10 cm apart at a stretching rate of 25 mm/min. Young's modulus and tensile strength were then calculated. (Sothornvit, 2009).

#### 2.3.4 Water vapor transmission rate (WVTR)

Water vapor transmission rate (WVTR) was measured by Farhadi and Javanmard (2021)’s method. In summary, the film was tested on a cup containing 12 ml of distilled water. Within 2 h, steady‐state conditions were assumed to have occurred. The cells were stored in a temperature‐ and humidity‐controlled room (a/c unit supplied by Denco Ltd.; conditions: 50 ± 5 percentage relative humidity, 23 ± 2°C), and a fan set at an air velocity of 154 mm/min was placed over the cells to ensure uniform movement of air. Eight weight measurements were then recorded over a 24‐h period with intervals of greater than 1.5 h between readings. Water vapor transmission rate was measured using the ASTM‐E 96 method (ASTM E96/E96 M‐16, 2016).

The WVTR was calculated according to Eq. (2).

(2) WVTR=W×X/A

where WVTR is the water vapor transmission rate (g H2O mm cm^−2^), *x* is the average thickness of the film (mm), and *A* is the permeation area (cm^2^).

#### 2.3.5 Scanning Electron Microscope (SEM)

Exterior surfaces of cups coated were examined with a scanning electron microscope (Seron Technology Model, AIS2100). The surface of the samples was coated with gold to prevent electric charge during the test and analyzed at 20 kV (Farhadi & Javanmard, 2021).

#### 2.3.6 Popcorn moisture content analysis

A certain amount of the sample was weighed and dried in an oven at 105°C until it reached a constant weight (Iran Institute of Standards and Industrial Research (INSO) 5656, 2018). The moisture content was calculated from the following equation:

$$\text{Moisture}\;\text{content}=\left(M_{2}‐M_{3}\right)/\left(M_{2}‐M_{1}\right)\times 100$$

*M*1 is the weight of the empty container (in grams) and *M*2 and *M*3 are the weight of the sample and the container before and after drying (in grams), respectively.

#### 2.3.7 Popcorn texture analysis

Popcorn texture stiffness test was performed using a compression test or compaction test and a histometer (Hounsfield model H5KS) with a cell load of 500 Newtons. During the compression test, the maximum force required to compress the popcorn was applied by the machine prop. For each test, the popcorn sample was loaded by a 40‐mm‐diameter stainless steel cylindrical prop. After giving the start command via computer, the loading agent pressed the sample at a constant speed of one millimeter per second. The endpoint was 8 mm and the test speed was 60 mm/min. The amount of force applied to the popcorn texture was measured and the texture stiffness was calculated and reported based on the force applied to the popcorn surface in Newtons (N) (Alipour et al., 2017).

#### 2.3.8 pH measurement

The pH of the popcorn samples was calculated using a pH meter (Metrohm model 691) according to the national standard of Iran, 5656. For this purpose, 2 g of the sample was dissolved in 20 ml of water and then the pH was read by placing a precalibrated pH meter electrode (Iran Institute of Standards and Industrial Research (INSO) 5656, 2018).

#### 2.3.9 Sensory evaluation

The method used in this research is the 5‐point hedonic method. In this method, for sensory evaluation, 19 evaluators who were randomly selected from different age groups, with different levels of education and including both sexes were selected. The treated samples were examined for taste, odor, appearance, and general acceptance. The test was defined as a 5‐point utility test (excellent, excellent, good, average, and bad) (Hassani et al., 2010).

### 2.4 Statistical analysis

Response surface methodology (RSM) is a collection of statistical and mathematical techniques for modeling and analysis of problems in which a response of interest is influenced by several variables (Montgomery, 2017). In other words, RSM is a statistical method that uses quantitative data from appropriate experiments to determine regression model equations and operating conditions (Mannan et al., 2007). A standard RSM design called central composite design (CCD) was applied in this work to study the variables in the coating process of a paper cup.

The central composite design was used for fitting a second‐order model. This method requires only a minimum number of experiments. Generally, these designs (CCD) consist of a 2*^n^* factorial or fraction (coded to the usual ±1 notation) augmented by 2*n* axial points (±α, 0, 0,..., 0), (0, ±α, 0,..., 0),..., (0, 0,..., ±α), and n_c_ center points (0, 0, 0,..., 0) (Myers et al., 2016). The center points were used to evaluate the experimental error and the reproducibility of the data. The Face Centered Central Composite Design is that all the axial points are projected on the surfaces so α = ±1. This design (Face Centered CCD) requires three levels of each factor.

The responses and the corresponding parameters are modeled and optimized using ANOVA to estimate the statistical parameters using response surface methods. This optimization process involves three major steps, which are, performing the statistically designed experiments, estimating the coefficients in a mathematical model, and predicting the response, and checking the adequacy of the model.

$$Y=f\left(X_{1}.X_{2}.\ldots .X_{n}\right)$$

where *Y* is the response variable and *X_i_* is the independent variables that are often called factors. The goal is to optimize the response variable (*Y*). It is assumed that the independent variables are continuous and controllable by experiments with negligible errors. It is required to find a suitable approximation for the true functional relationship between independent variables and the response surface (Gunaraj & Murugan, 1999).

The experimental sequence was randomized to minimize the effects of the uncontrolled factors. In this study, a central composite design was used to develop an empirical model and correlation between the variables of rice bran wax at three levels (0.2, 0.4, 0.6) and whey protein isolate at three levels (5.5, 7.75, 10) to the response variables (thickness (microns), color determination, Young's modulus (MPa), tensile strength (MPa), and moisture permeability (g/m^2^ day)) using a second‐order polynomial equation as given below:

$$Y=\beta_{0}+\sum_{i=1}^{n}\beta_{i}X_{i}+\sum_{i=1}^{n}\beta_{\mathrm{ii}}X_{i}^{2}+\sum \sum_{j<i}^{n}\beta_{\mathrm{ij}}X_{i}X_{j}$$

where *Y* is the predicted response, *β*_0_ the constant coefficient, *β*_1_ the linear coefficients, *β_ij_* the interaction coefficients, *β_ii_* the quadratic coefficients, and *x_i_*, *x_j_* are the coded values of the rice bran wax and whey protein isolate variables. The number of tests required for the CCD includes the standard 2*^n^* factorial with its origin at the center, 2*n* points fixed axially at a distance, say α from the center to generate the quadratic terms, and replicate tests at the center; where n is the number of variables. Hence, the total number of tests (*N*) required for the two independent variables is:

$$N=2^{n}+2n+n_{c}=2^{2}+\left(2\times 2\right)+3=11$$

Once the desired ranges of values of the variables are defined, they are coded to lie at ±1 for the factorial and axial points and 0 for the center point, as shown in Table 1.

The statistical software package Design‐Expert, version 11.1.1.0 (Stat‐Ease, Inc.) was used for regression analysis of experimental data to fit the equations developed and also to plot response surface. ANOVA (analysis of variance) was used to estimate the statistical parameters.

To evaluate the effect of the coated cup on moisture, pH, texture, and sensory evaluation of popcorn and compare it with the control sample, data were collected in three time intervals of 14, 28, and 42 days with three replications and two‐way ANOVA method was used. Also, the means were compared with Duncan's multiple range test and *T*‐test at a 5% probability level between different treatments. Data analysis in this section was performed using SPSS statistical software version 22.

## 3 RESULTS AND DISCUSSION

In this research, the response surface methodology of the central composite design of the center of the surface was used to optimize the coating process of simple popcorn cups. A second‐order model was fitted to each response variable. Regression analysis and ANOVA were performed to fit the model and determine the statistical significance of the model variables. The estimated coefficients from quadratic regression and ANOVA polynomial models are shown in Table 2, respectively. The positive coefficient indicates that as the value of the independent variable increases the mean of the dependent variable also tends to increase, and the negative sign is vice versa (Chen et al., 2008). Also, the value of the coefficients obtained based on the coded variables showed that the independent variable has the most effect on the response variable.

**TABLE 2:** Analysis of variance and estimated regression coefficients of second‐order polynomial models for response variables

Source	*df*	Coefficient Estimate					sum of squares					F‐Value				
		Thickness (µm)	∆E	Young's Modulus (MPa)	Tensile Strength (MPa(	WVTR (g/m^2^ day)	Thickness (µm)	∆E	Young's Modulus (MPa)	Tensile Strength (MPa(	WVTR )g/m^2^ day)	Thickness (µm)	∆E	Young's Modulus (MPa)	Tensile Strength (MPa(	WVTR (g/m^2^ day)
Model	5	+279.00	+4.32	+12.09	+11.19	+22.80	387.64	105.02	5.94	4.18	264.42	15.45**	17.45**	45.77**	23.90**	163.74***
ß_1_	1	+6.00	+2.14	−0.7383	−0.7050	−5.65	216.00	27.52	3.27	2.98	191.53	43.06**	22.87**	126.05***	85.24**	593.05***
ß_2_	1	+4.67	+2.94	−0.6200	−0.3183	+2.08	130.67	51.92	2.31	0.6080	25.92	26.05**	43.14**	88.88**	17.38**	80.25**
ß_12_	1	+2.75	+2.19	+0.2100	−0.0725	−0.2525	30.25	19.23	0.1764	0.0210	0.2550	6.03*	15.98*	6.80*	0.6010 ^ns^	0.7896^ns^
ß_11_	1	+1.0000	−0.9618	+0.2361	+0.4739	+3.99	2.53	2.34	0.1412	0.5691	40.28	0.5050^ns^	1.95 ^ns^	5.44 ^ns^	16.27 *	124.71**
ß_22_	1	−2.00	+1.47	−0.1889	−0.1261	+0.4724	10.13	5.46	0.0904	0.0403	0.5653	2.02 ^ns^	4.54 ^ns^	3.49^ns^	1.15 ^ns^	1.75^ns^
Residual	5						25.08	6.02	0.1297	0.1749	1.61					
Lack of Fit	3						12.42	5.79	0.0815	0.1171	1.48	0.6535^ns^	17.05^ns^	1.13^ns^	1.35^ns^	7.47^ns^
*R* ^2^		0.9392	0.9458	0.9786	0.9598	0.9939										
Adjusted *R* ^2^		0.8785	0.8916	0.9572	0.9197	0.9879										

### 3.1 Thickness

The results of ANOVA (Table 2) showed that the linear effect of rice bran wax and whey protein isolate on the thickness of the coated cup (*p* < .01) was significant. Besides, the interaction effect of rice bran wax and whey protein isolate was significant (*p* < .05). The effects of second‐order rice bran wax and whey protein isolate were not significant.

The positive sign of the regression coefficients obtained from the central composite design model (Table 2) was the sense of the direct effect of rice bran wax and whey protein isolate on the thickness of the coated cup. In other words, with increasing rice bran wax and whey protein isolate, the thickness of the coated cup increased. Also, the values of the coefficients indicated that the variable of rice bran wax had a greater effect on the thickness of the coated cup. A high value of *R* ^2^ indicates a better fit for the suitability of the obtained model.

**FIGURE 1:**
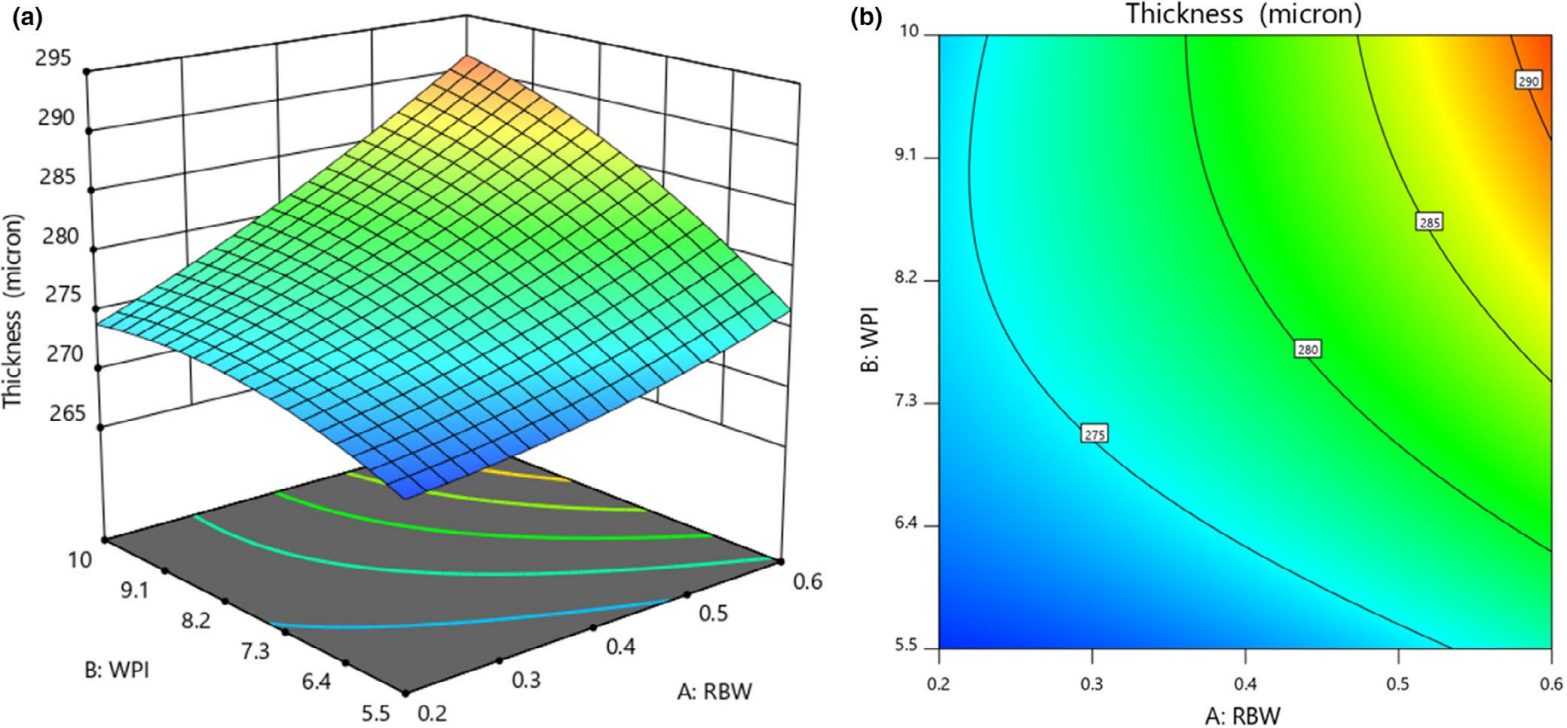
Response surface (a) and Contour (b) curves for the interaction effect of rice bran wax and whey protein isolate concentration on the thickness of coated cups

To visualize the interactions of the two independent variables with each response variable, contour curves and response surface procedures were created for each fitted model. Figure 1 shows the interaction effect of rice bran wax and whey protein isolate on the thickness of the coated cup using the response surface and contour curves, respectively. The highest thickness (293 microns) for rice bran wax with a concentration of 0.6 g and whey protein isolate with a concentration of 10 g and the lowest thickness (269 microns) for rice bran wax with a concentration of 0.2 g and whey protein isolate with a concentration of 0.5 5 g was observed. The effect of rice bran wax and whey protein isolate on the thickness of the coated cup, if the other variable is constant in its middle level, was such that with increasing rice bran wax (0.2 to 0.6 g) and whey protein isolate (5. 50 to 10 g) the thickness of the coated cup increased from 275 to 284 microns and from 273 to 280 microns, respectively, due to the increase in the concentration of the coating material. The thickness of the coatings is affected by the concentration of solids in the coating solution. Such a dependence of the coating thickness on the concentration of the coating solution has been observed in Kraft paper coated with other biopolymers. In general, the coating is affected by various factors such as the materials used to prepare the coating solution, coating temperature, drying temperature, and drying rate (Yu et al., 2006). Vrabič Brodnjak and Tihole (2020) developed new mixtures of coatings with zein, chitosan, and rosemary essential oil on paper with water and oil resistance. The coating thickness influenced the bending and tearing stiffness of the paper.

### 3.2 Color changes

The brightness index (L*), green index (a* negative), and yellowness index (b* positive) of uncoated paper cups were 94.44, −0.77, and 3.23, respectively. The coated cups L* index decreased compared to the amount of uncoated L* cups, which means that the coating made the cups darker. Han et al. (2001) concluded that the value of the L* index decreased significantly with increasing the amount of whey protein isolate, although this reduction was very small. Therefore, this difference may not be recognizable to the human eye, but it can be important in the process of printing on paper (Han & Krochta, 2001). The values of a* and b* in coated cups increased compared to the values of a* and b* in uncoated cups. The highest amount of total color change (∆*E*) in cups (11.84) with a concentration of 0.6 g of rice bran wax and a concentration of 10 g of whey protein isolate and the lowest amount (1.37) with a concentration of 0.2 g of rice bran wax and a concentration of 5.5 g of whey protein isolate was obtained. The results of ANOVA (Table 2) showed that rice bran wax and whey protein isolate (at 1% probability level) and their interaction (at 5% probability level) had a significant effect on the total color change (∆*E*) of the coating cup. Had. Also, the value of *R* ^2^ was 0.95. Positive coefficients (Table 2) indicated that with increasing concentrations of rice bran wax and whey protein isolate variables, total color change (∆*E*) increased. The coefficients also showed that whey protein isolate had a greater effect on the amount of total color change (∆*E*).

**FIGURE 2:**
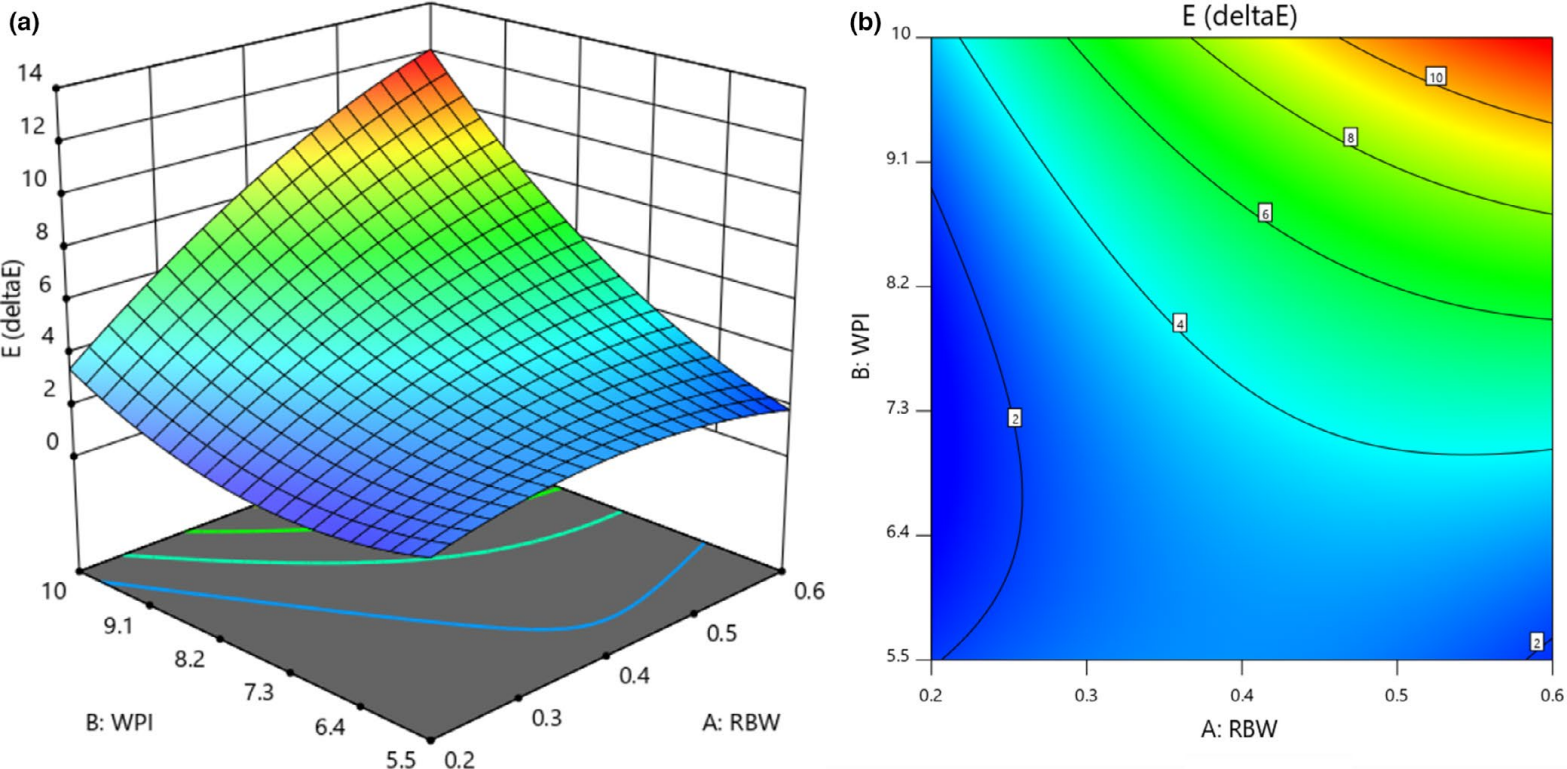
Response surface (a) and Contour (b) curves for the interaction effect of rice bran wax and whey protein isolate concentration on Color changes (∆E) of the coating cup

Figure 2 shows the interaction of rice bran wax and whey protein isolate with color changes in the coated cup. With the increasing concentration of rice bran wax and whey protein isolate, the amount of color changes increases due to the increase in concentration and inherent color of rice bran wax and whey protein isolate. Colorless packaging coatings are usually preferred by food manufacturers. Jiang et al. (2014) added fluorocarbons to a mixture of modified starch and sodium alginate to obtain a uniform coating solution, and then the solution was coated on a transparent paper surface. The color and barrier properties of the paper against oil were studied at different coating weights. The results showed that a*, b*, and L* of coated and uncoated papers were not significantly different.

### 3.3 Tensile properties

#### 3.3.1 Young's module

The effect of rice bran wax and whey protein isolate on the Young's modulus of coated cups is presented in Table 2. The results showed that rice bran wax and whey protein isolate (*p* < .01) and their interaction had a significant effect (*p* < .05). Also, the value of *R* ^2^ was 0.98. The negative sign of the estimated coefficients of Young's modulus shows the indirect effect of the independent variables of rice bran wax and whey protein isolate. Also, the estimated values of coefficients showed that rice bran wax had a greater effect on reducing Young's modulus amount than whey protein isolate.

**FIGURE 3:**
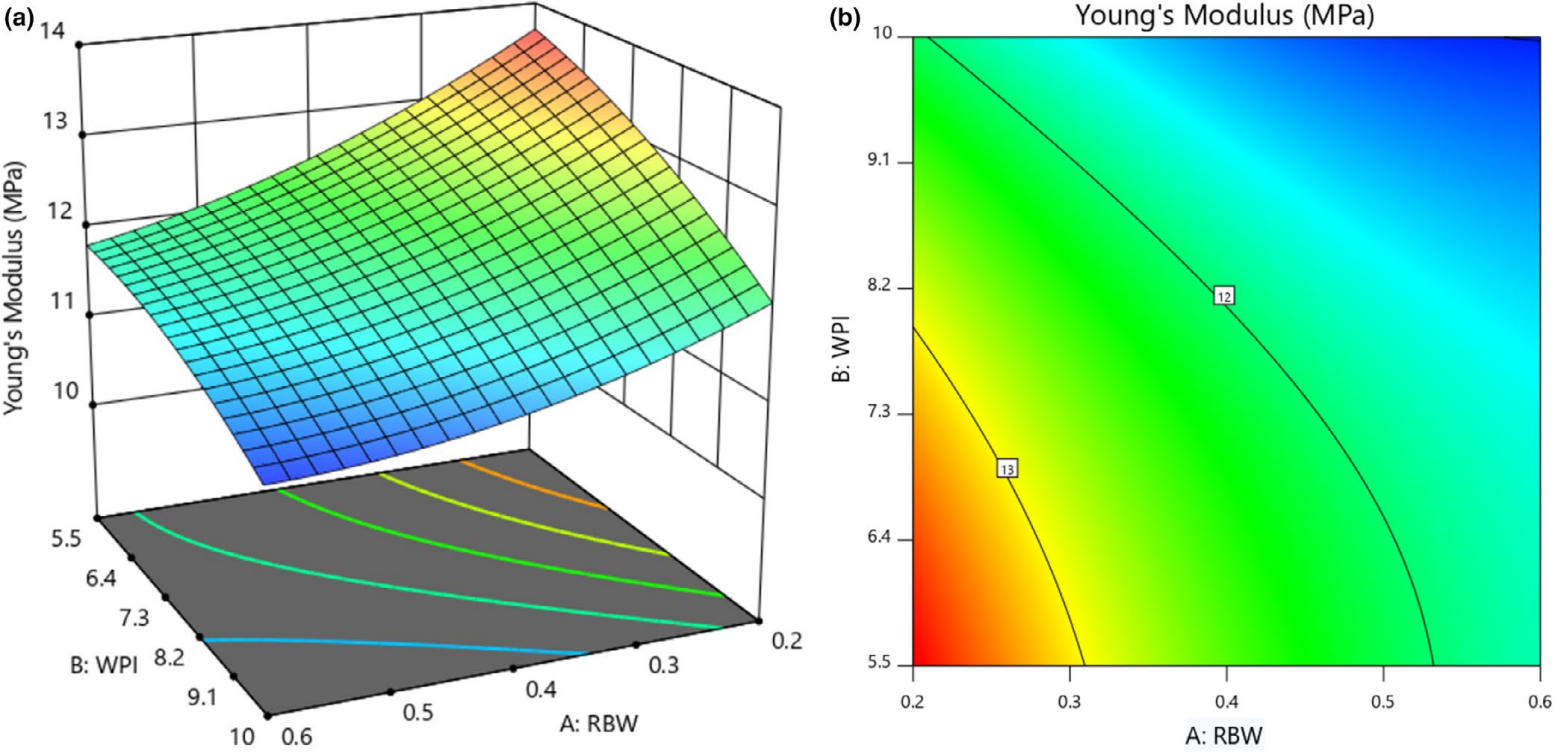
Response surface (a) and Contour (b) curves for the interaction effect of rice bran wax and whey protein isolate concentration on Young's modulus of coated cups

Figure 3 shows the effect of rice bran wax and whey protein isolate on Young's modulus of coated cup. According to this figure, with increasing the concentration of each of the two variables of whey protein isolate and rice bran wax, Young's modulus value of the coated cups decreased. Han and Krochta (1999) showed an increase in paper flexibility by whey protein isolate coating. Also, they found that during the coating process, a solution containing whey protein isolate swells cellulose and changes the structure of the paper fibers, and that the whey protein isolate penetrates the spaces between the paper fibers. After drying, whey isolate remains in the cellulose structure and interferes, and due to this interference, Young's modulus decreases

#### 3.3.2 Tensile strength

The effect of rice bran wax and whey protein isolate on the tensile strength of the coated cup is presented in Table 2. The results showed that rice bran wax and whey protein isolate had a significant effect on the amount of tensile strength (*p* < .01). Also, the value of *R* ^2^ was 0.95. The negative sign of the estimated coefficients of the model predicted for the tensile strength of the coated cup indicates that the amount of tensile strength was indirectly related to the independent variables of rice bran wax and whey protein isolate. Also, the estimated coefficients showed that rice bran wax had a greater effect on reducing the tensile strength of the coated cup than whey protein isolate.

**FIGURE 4:**
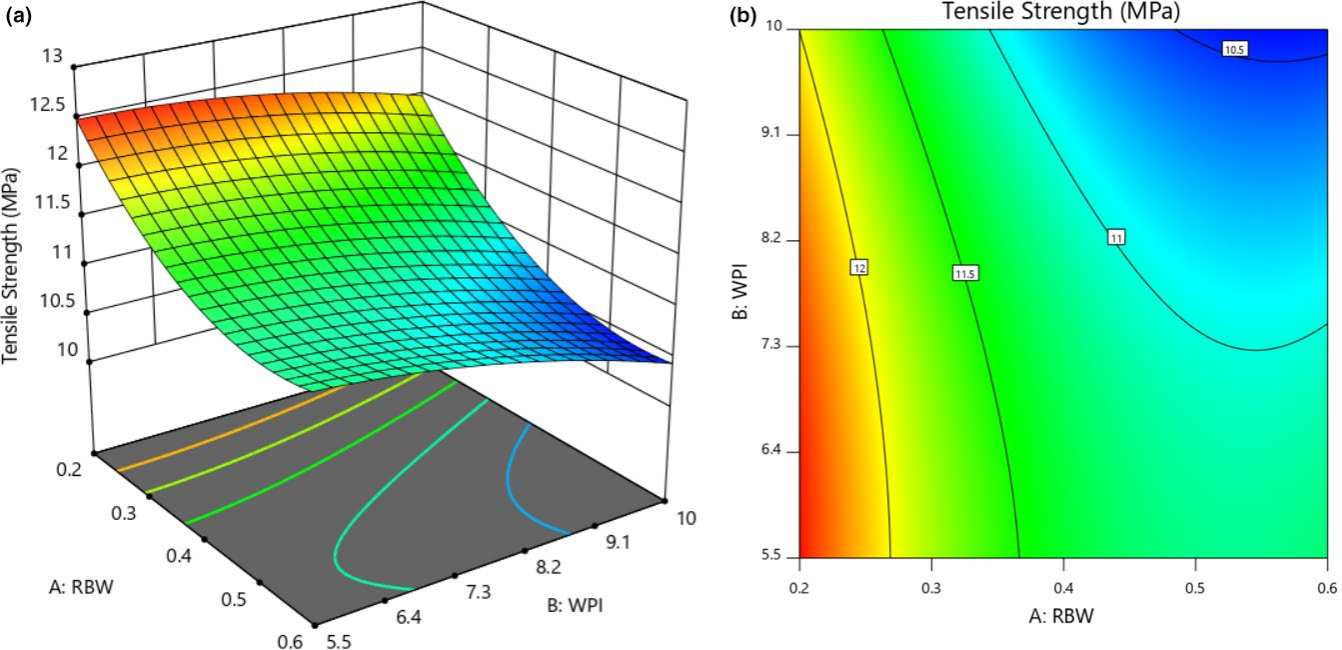
Response surface (a) and Contour (b) curves for the interaction effect of rice bran wax and whey protein isolate concentration on the tensile strength of coated cups

Figure 4 shows the effect of rice bran wax and whey protein isolate on the tensile strength of the coated cup. According to this figure, with increasing the concentration of each of the two variables of whey protein isolate and rice bran wax, tensile strength of the coated cups decreased. Decreased tensile strength in the paper indicates a decrease in the cardboard's ability to break under stress. Rhim et al. (2006) showed that the reduction in tensile strength of the coated paper was due to a decrease in the strength of the cellulose fibers of the paper, which may be due to the penetration of the coating material into the paper structure. The fibers and their layers are connected by fiber bonds, which transmit shear forces and other network loads. The amount and size of the fiber bonds depend on the internal fibrillation, which also influences fiber swelling, elongation, and flexibility (Vrabič Brodnjak & Tihole, 2020).

### 3.4 Water vapor transmission rate

Table 2 shows that rice bran wax (*p* < .01) had a significant effect on the water vapor transmission rate in the coated cup. Whey protein isolate and the interaction effect of rice bran wax and whey protein isolate was not significant (*p* > .01). Also, the value of *R* ^2^ was 0.99. This value indicated that the predicted model was suitable for predicting the degree of WVTR. Response and level surface curves were created using significant variables for the degree of moisture permeability in the coated cup.

**FIGURE 5:**
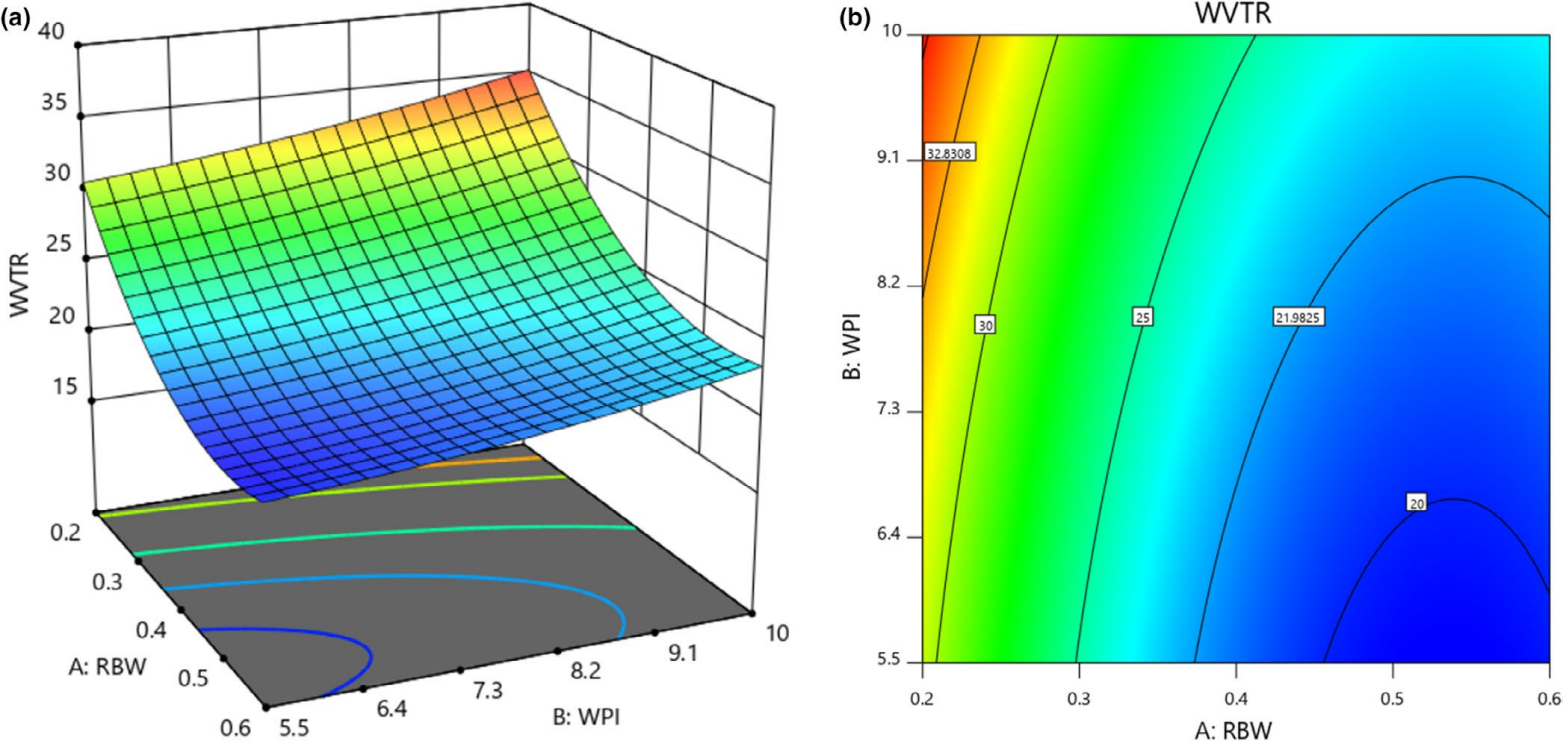
Response surface (a) and Contour (b) curves for the interaction effect of rice bran wax and whey protein isolate concentration on the WVTR of coated cups

Figure 5 shows the interaction of rice bran wax and whey protein isolate for water vapor transmission rate in the coated cup. According to the figure, the highest water vapor transmission rate (32.83 g/m^2^ day) was in the concentration range of 0.2–0.25 g of rice bran wax and in the concentration range of 7.75–10 g of whey protein isolate. The lowest water vapor transmission rate (21.98 g/m^2^ day) was obtained in the concentration range of 3.34–0.6 g of rice bran wax and the concentration range of 5.5–10 g of whey protein isolate. From this figure, it can be concluded that by increasing the concentration of rice bran wax and decreasing the concentration of whey protein isolate, the WVTR decreased, significantly. The hydrophilic properties of natural polymers such as proteins cause a decrease in the vapor barrier properties (Ismaili et al., 2014). Water permeability is directly related to the number of hydroxyl groups in the molecule (Katz & Labuza, 1981). Thickness created by rice bran wax is another reason for reducing the amount of water vapor transmission in coated cups. The reason for increasing the amount of water vapor transfusion in the range of 0.2–0.25 g of wax rice bran and in the range of 7.75–10 grams of whey protein can be related to cracks that are covered during the coating process. The WVTR could generally be affected by the hydrophilic part of the coating and depends on the hydrophilic–hydrophobic ratio of the coating components (Norajit et al., 2010). The advantage of coated paper is that it reduces water vapor permeability. The decrease in the weight of whey protein‐coated papers compared to soybean protein and corn rye protein is due to differences in the type of coating biopolymer used, indicating differences in the interaction between the biopolymer and cellulose fiber structures as a result of changes in water vapor permeability (Han et al., 2010; Rhim et al., 2006). In this study, we selected whey protein isolate due to its inhibitory properties and soft tissue formation on the outer surface of the cup. Hamdani et al. (2020) showed that the application of a zein coating onto paper resulted in a reduced WVTR, which corresponds to the masking of the pores by zein as well as the moderate hydrophobicity of zein. Khwaldia et al. (2010) compared uncoated paper with paper coated with sodium caseinate and carnauba wax and showed that the coating improved the water‐repellent properties of the paper (Khwaldia et al.,^2010^).

Uncoated paper compared with zein‐, chitosan‐, and rosemary essential oil‐coated paper has a hydrophilic nature, and due to its porous structure, it has a high capability of water and moisture uptake (Vrabič Brodnjak & Tihole, 2020).

### 3.5 Microstructure of coated cups by scanning electron microscope (SEM)

**FIGURE 6:**
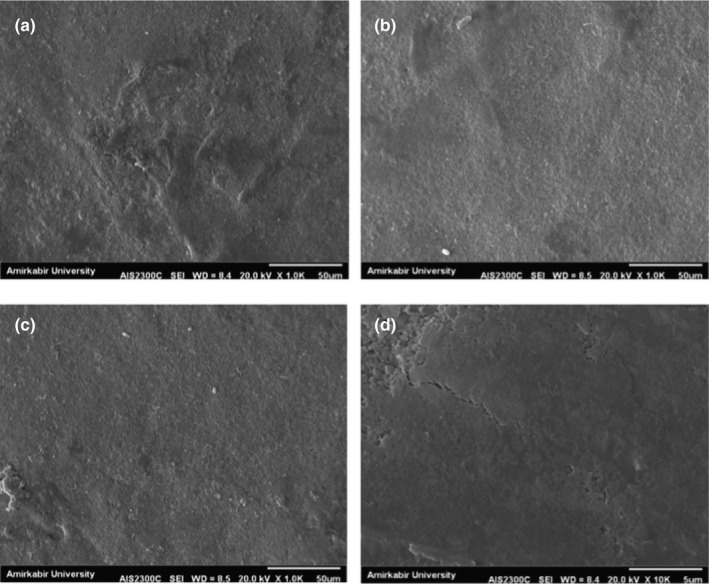
Scanning electron micrograph (SEM) images of the outer surface views of paper cups (a) coated with 0.2 g rice bran wax and 5.5 g whey protein isolate, (b) coated with 0.6 g rice bran wax and 10 g whey protein isolate, (c) coated with 0.4 g rice bran wax and 7.75 g whey protein isolate, and (a) uncoated paper cup

Figure 6 shows the SEM micrographs from the surface of coated cups treated with different formulations (rice bran wax 0.2 g and whey protein isolate 5.5 g, rice bran wax 0.6 g and whey protein isolate 10 g, rice bran wax 0.4 g and whey protein isolate 7.75 g, and control sample) and the uncoated paper cup. The uncoated paper cup's external surface has a porous structure. These pores on the surface of the paper cause the paper to become permeable. The coated paper cups showed a smooth surface and the coating fills the pores of the surface, increasing the barrier properties of the paper and also reducing the roughness.

The results of Han et al. (1999) showed that the presence of a coating on the paper surface improves the inhibitory properties compared to the control sample. In Figure 6, in comparison with the control sample (d) and the other three treatments, it is clear that the coating has caused the disappearance of cavities. Hamdani et al. (2020) showed that paper coated with zein, CHI, CHI−g−PDMS, and CHI−g−PDMS/zein showed significantly reduced surface porosity in comparison with uncoated paper. Han and Krochta (2001) showed that increasing the concentration of whey protein isolate makes the paper surface smoother. As shown in Figure 6 (b), by increasing the concentration of whey protein isolate, the surface of the paper cup has become smoother, too. Buck et al. (2015) examined the morphological structure of rice bran wax. The results showed that rice bran wax has large, heterogeneous needle crystals. As the wax concentration increases, the dispersion of the crystals decreases, which in turn increases the inhibitory properties of the coating. Figure 6(a), comparing two treatments (b) and (c), shows that the dispersion of rice bran wax‐coated surface has increased with increasing wax concentration in coating formulation.

### 3.6 Optimization

The optimal operating conditions for all variables using the numerical optimization technique of Design Expert software (Design‐Expert software version 11) are expressed in Table (3). This optimization is in order to achieve coated cup and the optimal coating conditions of the cups for use in food products. The values of WVTR, Young's modulus, thickness, color changes, and tensile strength were 19.785 (g/m^2^ day), 11.810 (MPa), 276.583 (µm), 1.839, and 11.222 (MPa), respectively.

**TABLE 3:** Optimization results of cup coated with rice bran wax and whey protein isolate by response surface methodology (WVTR, TS, YM, ∆E, and Thickness)

Number	Rice Bran Wax (g)	Whey Protein Isolate (g)	Thickness (μm)	∆E	Youngs's Modulus (MPa)	Tensile Strength (MPa)	WVTR (g/m^2^ day)	Desirability
1	0.600	5.500	276.583	1.839	11.810	11.222	19.785	0.699
2	0.600	5.532	276.583	1.870	11.810	11.220	19.798	0.697
3	0.515	5.500	276.583	2.501	12.054	11.174	19.408	0.681
4	0.495	5.500	276.583	2.611	12.126	11.187	19.533	0.678
5	0.470	5.500	276.583	2.716	12.220	11.217	19.794	0.675
6	0.466	5.500	276.583	2.731	12.236	11.224	19.851	0.674
7	0.430	5.500	276.583	2.822	12.386	11.296	20.475	0.668
8	0.399	5.500	276.583	2.851	12.528	11.384	21.228	0.661

### 3.7 Popcorn moisture

According to Table 4, the results showed that with increasing storage time, the moisture content absorption increased, which was more in the control sample than in the treatment sample, but this increase was not significant (*p* < .05). Also, the coating formulation did not significantly affect moisture absorption of popcorn (*p* < .05). The increase in the moisture content of popcorn is due to the type of packaging materials and the type of capping of the cups during storage of popcorn. Upon popping, corn kernels undergo substantial dehydration. The initial moisture content of the kernel, popping method, and temperature determine the final quality and physical characteristics of the flakes (García‐Pinilla et al., 2021). The type of material and packaging method is very effective in the moisture absorption of popcorn (Cheng, 2005). The amount of moisture absorbed in the product through packaging has a significant effect on products with low humidity such as popcorn compared to changes due to moisture.

**TABLE 4:** Physicochemical analysis of packaged popcorn in coated and uncoated cups during storage time

Treatment	Day 14			Day 28			Day 42		
	Moisture	Texture	pH	Moisture	Texture	pH	Moisture	Texture	pH
Uncoated cup	4.83 ± 0.29^aA^	12.24 ± 2.05^aA^	5.717 ± 0.05^aA^	5.00 ± 0.00^aA^	13.63 ± 1.06^aA^	5.6 ± 0.04^aA^	5.33 ± 0.57^aA^	15.61 ± 0.38^bA^	5.34 ± 0.06^bA^
Coated cup	4.50 ± 0.00^aA^	11.16 ± 1.40^aB^	5.73 ± 0.08^aA^	4.67 ± 0.29^aA^	11.76 ± 1.60^aB^	5.69 ± 0.09^aA^	5.0 ± 0.87^aA^	14.30 ± 0.340^bB^	5.48 ± 0.17^bA^

Note

1. Values are reported as means of three replicates ±Standard Deviation (SD).

2. Different lowercase and uppercase are shown the significant difference (*p* < .05) between storage time and samples, respectively.

### 3.8 Popcorn texture

According to Table 4, the results showed that increasing the storage time increased the stiffness of the popcorn tissue significantly (*p* <.05). Also, a significant decrease in brittleness and increase in the firmness of the popcorn was attained from the control sample in comparison with the treated sample. With increasing moisture content, the food changes from brittle to viscoelastic (Van Hecke et al., 1998). Coated cups had a lower WVTR affecting the moisture content of popcorn (Figure 5). Increasing the moisture content of uncoated packaged popcorn causes the popcorn to shrink and become sticky, thus reducing the crispness of the product. Therefore, changes in humidity affect the physical and mechanical properties of popcorn and the main and effective factor in the crumbliness of popcorn at consumption depends on the type of popcorn packaging material.

These results were similar to Cheng's (2005). The results of Katz and Labuza (1981) showed that dry and crunchy snacks, such as potato chips, popcorn, and crackers, usually lose their crispness by absorbing moisture in the range of 0.35–0.5. The optimal texture of food products, especially between meals, is sensitive to increased humidity. Increasing aw between dry snacks reduces sensory acceptance (Katz & Labuza, 1981).

### 3.9 pH

The data in Table 4 give the pH of popcorn for storage time and coating formulations. The results showed that increasing the storage time caused a significant decrease in the pH of the treatment and control sample (*p* < .05) (Table 4). However, coating formulations did not significantly affect the pH of popcorn (*p* > .05). pH decreasing in control was slightly greater than in the treatment. pH decreasing is probably due to the activity of microorganisms and the production of various organic acids. The rate of pH reduction during storage in food packaging depends on the storage temperature, packaging method, organic acid content, and the amount of carbon dioxide in them (Mousavian et al., 2021).

### 3.10 Sensory evaluation of popcorn

Mean sensory attribute scores and standard deviations for the effects of storage time and coating are given in Table 5. Popcorn taste was significantly affected by storage time (*p* < .01) and cups’ coating formulations (*p* < .01). Results showed that with increasing storage time, popcorn taste decreased (Table 5). Taste is closely associated with aroma, and many compounds have been identified that contribute to popcorn aroma and flavor, including pyrazines, furans, pyrroles, carbonyls, and substituted phenols, with pyrazines playing a major role in imparting the characteristic popcorn flavor and aroma (Sweley et al., 2013).. Popcorn odor scores packed in the uncoated cup was higher than the ones in the coated cup (Table 5). Grosch & Schieberle (1997) showed that the taste of popcorn is not stable. Decreasing the concentrations of 6‐acetyltetrahydropyridine and 2‐propionyl‐1‐pyrroline during 7‐day storage reduces the quality of popcorn flavor. Shibaleh et al. (1995) found that freshly roasted corn had lower levels of 2‐propionyl 1‐1 pyrroline than 6‐acetyltetrahydropyridine. Concentrations of 2‐propionyl‐1‐pyrroline and 6‐acetyltetrahydropyridine decreased by about one‐third during the storage of the popcorn sample in a polyethylene bag for 1 week.

**TABLE 5:** Scores mean ± SD of sensory evaluation for popcorns packaged in coated and uncoated cup

Score Attribute	Coated cup	Uncoated cup
Taste	3.84 ± 0.33^a^	3.05 ± 0.18^b^
Odor	4.34 ± 0.22^a^	4.18 ± 0.13^a^
Appearance	4.22 ± 0.62^a^	4.16 ± 0.48^a^
General acceptance	3.43 ± 0.48^a^	3.26 ± 0.32^a^

The results showed that storage time and coating formulation has no significant effect on popcorn odor (Table 5). Pyrazines, furans, pyrroles, carbonyls, and phenols play an important role in creating the taste and smell of popcorn (Walradt et al., 1970). Low pH reduces the Millard reaction, which is the main cause of popcorn odor and taste, and thus reduces the popcorn flavor and odor during storage. Walradt et al. (1970) identified a total of 58 volatiles in an extract of microwave‐popped corn. Schieberle (1991) indicated that 2‐acetyl‐l‐pyrroline, (E$)‐ 2,4‐decadienal, 2‐furfurylthiol, and 4‐vinyl‐kmethoxypheno1 were the most important odorants in fresh popcorn.

Popcorn appearance showed no significant effect by storage time and coating formulation (*p* < .05). (Table 5).

Overall acceptability based on raw taste and texture of popcorn and desirability of general acceptance by panelists showed that there was a significant decrease during storage (*p* > .05). In other words, with increasing time, overall acceptability of popcorn decreased. The general acceptance score of packaged popcorn in coated cups was higher than popcorns in uncoated cups. Increasing the humidity and decreasing the volume reduced the brittleness of the product during the storage period and as a result reduced the general acceptance of the samples (Table 5). High‐quality popcorn texture is defined as crispy and melts in the mouth, versus low quality being that which is chewy and adhesive to the teeth (Sweley et al., 2013).

Plimpton (1984) showed that packaging materials had significant effects the popcorn shelf life stability, off flavor, rancidity development, and crispness deterioration.

Popcorns packed in the uncoated cups could be expected to lose odor, flavor, and decrease in crispiness and overall acceptability more than popcorns packed in the coated cups.

## 4 CONCLUSION

Whey protein isolate and rice bran wax showed a good potential as biodegradable edible coating materials for paper cup packaging. Paper cups’ mechanical, moisture barrier properties and glossy appearance coated with whey protein isolate and rice bran wax were improved. Based on the results of this study, the coating containing 0.6% rice bran wax and 5.5% whey protein isolate on the outer surface was recognized as the best coating formulation. Also, the surface morphology of coated cups showed that coating eliminates surface pores. Whey protein isolate and rice bran wax in coating formulation caused a smoother surface in cups. The results showed that packing popcorn with coated cups during storage reduces the pH and moisture absorption of popcorn compared to the samples packed in uncoated cups. Biodegradable coating of paper cups and packaging papers could be a new approach for environmentally friendly and biodegradable packages for shelf life improvement of packed dried products, snacks, and also for moisture‐susceptible products.

## CONFLICT OF INTEREST

The authors declare no conflict of interest.

## ETHICAL APPROVAL

This study was approved by the ethical guidelines for biomedical research involving human subjects number 11471/P/1 issued in January 1992 by the Ministry of Health and Medical Education of Iran.

## Data Availability

The data that support the findings of this study are available on request from the corresponding author. The data are not publicly available due to privacy or ethical restrictions.
